# The Inflammatory Cell Death in Diabetic Kidney Disease: Integrating Multifactorial Mechanisms into Novel Therapeutics

**DOI:** 10.3390/ijms262211033

**Published:** 2025-11-14

**Authors:** Bin Fang, Wei Huang, Sijia Du, Yu Hao, Fangfang He, Chun Zhang

**Affiliations:** Department of Nephrology, Union Hospital, Tongji Medical College, Huazhong University of Science and Technology, Wuhan 430022, China; drfangbb@163.com (B.F.); hw15861341084@163.com (W.H.); dusijia_327@163.com (S.D.); timely_rain0504@163.com (Y.H.)

**Keywords:** diabetic kidney disease, inflammatory cell death, pathogenesis, inflammatory response, therapeutic strategies

## Abstract

In addition to apoptosis, inflammatory cell death modalities—including pyroptosis, necroptosis, ferroptosis, NETosis, and the integrated paradigm of PANoptosis—are now established as critical drivers of diabetic kidney disease (DKD) pathogenesis. This review summarizes how key inflammatory cell death molecular mediators—such as the NLRP3 inflammasome, the RIPK1/RIPK3/MLKL axis, executioner caspases, and gasdermin-D (GSDMD)—orchestrate the death of renal cells (podocytes, tubular cells, mesangial cells, endothelium), thereby propagating inflammation and fibrosis. Preclinical studies have demonstrated the efficacy of agents targeting these pathways, highlighting their therapeutic potential. Key challenges include achieving cell type-specific targeting, overcoming redundancy among cell death pathways, and improving the translational applicability of current models. Emerging solutions include the development of precise biomarkers, kidney-targeted delivery systems, and combination therapies that concurrently target multiple cell death axes. This review synthesizes evidence establishing inflammatory cell death as a cornerstone of DKD pathology and provides a conceptual framework to guide future research and therapeutic innovation.

## 1. Introduction

Diabetic kidney disease (DKD) is a severe complication of diabetes mellitus and a leading cause of end-stage renal disease (ESRD) worldwide, with its prevalence increasing alongside the global increase in diabetes incidence [1,2]. Epidemiological data indicate that DKD affects ~30–40% of individuals with type 1 diabetes and 20–40% of those with type 2 diabetes, imposing a considerable public health burden [3,4]. The pathogenesis of DKD involves hyperglycemia, oxidative stress, activation of the renin–angiotensin system (RAS), and the accumulation of advanced glycation end products (AGEs) [5,6,7], which collectively drive renal inflammation, fibrosis, and nephron loss. Inflammation is now recognized not merely as a downstream consequence but also as a central driver of DKD progression, making it a promising therapeutic target [8,9,10]. While early studies focused primarily on apoptosis—a regulated cell death pathway characterized by cellular shrinkage, nuclear fragmentation, and apoptotic body formation [11,12]—its contribution alone cannot fully explain the inflammatory cascades and fibrotic remodeling that aggravate kidney injury in DKD. Increasing evidence suggests that other inflammatory cell death modalities, including pyroptosis, necroptosis, ferroptosis, NETosis, and PANoptosis, promote inflammation by releasing damage-associated molecular patterns (DAMPs), thereby amplifying tissue injury [13,14,15,16,17,18]. This review synthesizes recent advances in understanding the role of inflammatory cell death in DKD and discusses its therapeutic implications.

## 2. Inflammatory Cell Death: From Molecular Mechanisms to DKD

Inflammatory cell death is a specific type of programmed cell death characterized by the release of proinflammatory cytokines and their intracellular contents, which can exacerbate tissue injury and contribute to a wide range of inflammatory diseases [19,20,21]. Unlike traditional apoptosis, apoptosis is defined by its pronounced inflammatory nature and distinct initiating mechanisms. On the basis of underlying molecular pathways and morphological characteristics, inflammatory cell death can be classified into several major types, including pyroptosis, necroptosis, ferroptosis, NETosis, and PANoptosis [22,23].

### 2.1. Pyroptosis: NLRP3 Inflammasome Activation in Diabetic Kidneys

Pyroptosis is an inflammatory form of programmed cell death executed by the canonical NLRP3 inflammasome pathway, wherein activated caspase-1 cleaves gasdermin D (GSDMD) to form plasma membrane pores, leading to cell lysis and the release of mature interleukin (IL)-1β and IL-18 [24,25]. In the diabetic kidney, this pathway is hyperactivated by a triad of key pathological triggers: hyperglycemia, the accumulation of advanced glycation end products (AGEs), and oxidative stress [26]. These stimuli converge to promote NLRP3 oligomerization and caspase-1 activation. Evidence from both diabetic mouse models and renal biopsies of patients with DKD consistently revealed upregulated NLRP3 expression and enhanced caspase-1 activity, underscoring the clinical relevance of this pathway [27,28]. Consequently, pyroptosis not only causes direct cellular lysis but also, more critically, the released IL-1β acts as a potent driver of both tubular and glomerular injury [29,30], thereby establishing a vicious cycle of inflammation and cell death that propagates kidney damage.

### 2.2. Necroptosis: RIPK1/RIPK3/MLKL Signaling and Sterile Inflammation

Necroptosis represents a proinflammatory form of regulated cell death orchestrated by the RIPK1/RIPK3/MLKL signaling axis [31]. In the context of DKD, upregulated TNF-α signaling serves as a primary trigger for this pathway [32]. A critical molecular switch occurs when caspase-8 activity is inhibited [33], redirecting cell fate from apoptosis to necroptosis. This leads to RIPK3-mediated phosphorylation of MLKL, its oligomerization, and subsequent plasma membrane disruption. The lytic outcome results in the release of damage-associated molecular patterns (DAMPs), such as HMGB1, which potently drive inflammation by activating innate immune receptors on neighboring cells [34]. Like pyroptosis, necroptosis amplifies inflammatory cascades through DAMP release; however, its distinct signaling machinery provides an independent and promising set of druggable targets for intervention in DKD progression.

### 2.3. Ferroptosis: Metabolic Dysregulation and Lipid Peroxidation in Diabetic Renal Injury

Ferroptosis is an iron-dependent form of regulated cell death driven by the lethal accumulation of lipid peroxides. Its core regulatory machinery involves a delicate balance between antioxidant defense and pro-oxidant drivers. The key inhibitory pathway is governed by glutathione peroxidase 4 (GPX4), which utilizes glutathione (GSH) to reduce lipid hydroperoxides to nontoxic alcohols [35,36]. Conversely, the promotive pathway is mediated by acyl-CoA synthetase long-chain family member 4 (ACSL4), which esterifies polyunsaturated fatty acids, increasing their susceptibility to peroxidation. An emerging parallel inhibitory system is orchestrated by ferroptosis suppressor protein 1 (FSP1), which functions independently of GPX4 by regenerating reduced coenzyme Q10 in the plasma membrane to act as a lipophilic radical-trapping antioxidant [37,38].

In DKD, this pathway is specifically activated by diabetic metabolic derangements [39]. Iron overload—facilitated by the upregulation of transferrin receptor 1 (TFR1)—and a state of high oxidative stress provide the fundamental “fuel” and “spark” for ferroptosis. Key molecular events include the downregulation of GPX4 and SLC7A11 (a core component of system Xc^−^) by hyperglycemia and inflammatory cytokines (e.g., TGF-β), crippling cellular antioxidant defenses [40]. Additionally, FSP1 has been implicated in the pathogenesis of DKD [41]. Concurrently, the upregulation of ACSL4 increases cellular susceptibility to lipid peroxidation [42]. The primary cells affected are metabolically active renal tubular epithelial cells. Ferroptotic death in these cells causes acute tubular injury and releases proinflammatory and profibrotic signals, such as the lipid peroxide 4-hydroxynonenal (4-HNE), which directly activates fibroblasts and drives the progression of tubulointerstitial fibrosis [43,44,45].

### 2.4. PANoptosis: An Integrated Cell Death Circuit in Podocyte Demise

PANoptosis represents a converging mechanism in DKD, integrating pyroptosis, apoptosis, and necroptosis to exacerbate podocyte loss and inflammation, with TNF-related apoptosis-inducing ligand (TRAIL) signaling as a key trigger. PANoptosis integrates pyroptosis, apoptosis, and necroptosis into a unified death program activated by infections and cellular stress [46]. In kidney diseases, including DKD, this convergence amplifies inflammation and injury. TRAIL has been identified as a PANoptosis mediator in podocytes, and targeting TRAIL may be therapeutic. PANoptosis interacts with ferroptosis and pyroptosis, compounding injury; ferroptosis-induced tubular cell death and pyroptosis-induced inflammation can be synergistically harmful when integrated into PANoptosis [18,47,48].

### 2.5. NETosis: Neutrophil Extracellular Traps in Glomerular Damage and Inflammation

NETosis is increasingly recognized in DKD pathogenesis, where neutrophil-derived extracellular traps (NETs) promote glomerular endothelial damage and thrombo-inflammation. NETosis is a neutrophil-specific cell death process that releases NET-DNA fibers coated with antimicrobial proteins—which, while protective against microbes, can cause collateral tissue damage when dysregulated [49]. In DKD, NETs accumulate in glomeruli, which is correlated with disease severity [50]. NET deposition damages glomerular endothelial cells, partly through pyroptosis. Degrading NETs with DNase I or inhibiting peptidyl arginine deiminase 4 (PAD4) reduces glomerular injury. NETs also activate the NLRP3 inflammasome, perpetuating IL-1β/IL-18 production, immune cell recruitment, fibrosis, and functional decline [51] (Table 1).

This table systematically compares five distinct forms of inflammatory cell death implicated in the pathogenesis of DKD: pyroptosis, necroptosis, ferroptosis, NETosis, and PANoptosis. For each type, core molecular mediators, key effectors/DAMPs released, primary renal cellular targets in dkd and potential biomarkers were listed. These pathways collectively promote renal damage through mechanisms such as the release of proinflammatory cytokines (e.g., IL-1β and IL-18), damage-associated molecular patterns (DAMPs), oxidative stress, neutrophil extracellular traps (NETs), and fibrotic responses. Understanding the unique and overlapping features of these cell death modalities provides insight into potential therapeutic targets for attenuating inflammation and cellular damage in DKD.

### 2.6. Crosstalk and Therapeutic Implications

These inflammatory cell death pathways do not operate in isolation but engage in extensive crosstalk [52,53]. For example, necroptosis-derived DAMPs (e.g., HMGB1) can activate the NLRP3 inflammasome to promote pyroptosis [54], whereas ferroptotic stress can potentiate both necroptotic and pyroptotic signaling [55]. This interplay creates a self-amplifying cycle of cell death and inflammation that accelerates DKD progression. The integrated understanding of these mechanisms reveals multiple therapeutic targets, with combination strategies simultaneously addressing multiple pathways potentially offering superior efficacy compared with single-pathway inhibition [56,57].

## 3. The Role of Inflammatory Cell Death in Diabetic Kidney Disease

**Figure 1:**
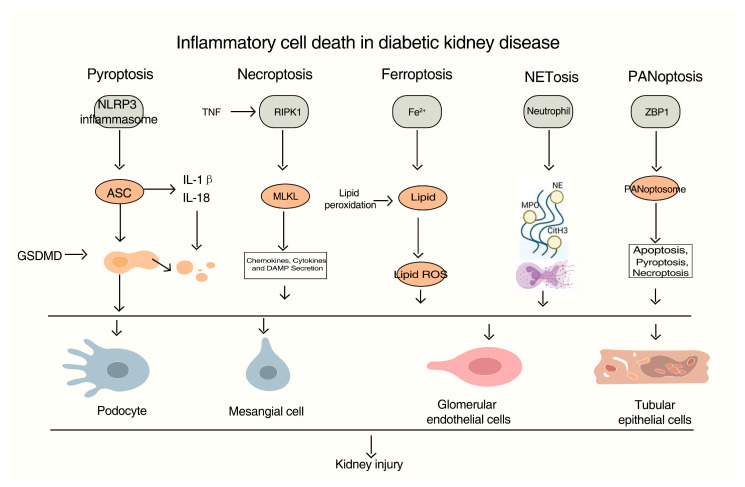
Mechanisms of inflammatory cell death in diabetic kidney disease (DKD).

The pathogenesis of DKD is complex and multifactorial, with inflammation serving as a pivotal driver of disease progression. Here, we summarize the role of inflammatory cell death in DKD (Figure 1).

This figure illustrates the key mechanisms of inflammatory cell death pathways implicated in diabetic kidney disease (DKD), including pyroptosis, necroptosis, ferroptosis, NETosis, and PANoptosis. Pyroptosis is mediated through the NLRP3 inflammasome, leading to the activation of ASC and the subsequent release of IL-1β and IL-18. Necroptosis is initiated by TNF signaling via RIPK1 and MLKL, resulting in lipid peroxidation and the secretion of chemokines, cytokines, and damage-associated molecular patterns (DAMPs). Ferroptosis is an iron-dependent (Fe^2+^) form of cell death driven by excessive lipid peroxidation, which damages tubular and endothelial cells. NETosis involves the release of neutrophil extracellular traps (NETs) containing proteases such as neutrophil elastase (NE) and myeloperoxidase (MPO), promoting inflammation and tissue damage. PANoptosis is a coordinated inflammatory cell death process that involves pyroptosis, necroptosis, and apoptosis. These pathogenic mechanisms collectively target key renal cells—including podocytes, mesangial cells, glomerular endothelial cells, and tubular epithelial cells—driving progressive kidney injury and the pathophysiology of DKD. These processes are central to the pathophysiology of DKD, linking metabolic and immune mechanisms to progressive renal damage.

### 3.1. Inflammatory Cell Death and Tubular Cell Injury

Research indicates that tubular epithelial cells (TECs) are particularly vulnerable to pyroptosis, necroptosis and ferroptosis under hyperglycemic conditions [58,59]. High glucose levels induce cellular stress, activating the NLRP3 inflammasome, a critical mediator of pyroptosis in DKD. This activation is triggered by factors such as oxidative stress and the accumulation of AGEs, both of which are prevalent in diabetic conditions. Studies have shown that hyperglycemia increases proinflammatory cytokine expression and activates caspase-1, which cleaves GSDMD, thereby facilitating pyroptosis in TECs [15,60]. Moreover, albuminuria further exacerbates TEC injury by promoting inflammatory responses [61]. Albumin and other proteins can activate toll-like receptors (TLRs) on TECs, increasing inflammatory mediator secretion and further activating the NLRP3 inflammasome. This creates a vicious cycle in which inflammation drives further cell death, contributing to DKD progression [16].

In addition to pyroptosis, necroptosis has also been implicated in renal tubular injury in DKD. Yu et al. reported that treatment with the RIPK1 inhibitor RIPA-56 suppressed necroptosis activation, reduced necroinflammation, and alleviated lipid accumulation, thereby improving renal outcomes [62]. Other studies have shown that targeting these pathways, including the inhibition of the NLRP3 inflammasome or the modulation of caspase activity, may offer new avenues for DKD treatment [63].

High glucose can also induce ferroptosis in renal tubular epithelial cells by disrupting iron homeostasis and promoting oxidative stress [64]. This process is mediated by the upregulation of transferrin receptor 1 (TFR-1), which increases the level of intracellular iron, and the downregulation of ferroptosis inhibitors, such as glutathione peroxidase 4 (GPX4), ferritin heavy chain 1 (FTH-1), and the cystine/glutamate antiporter solute carrier family 7 member 11 (SLC7A11). The resulting lipid peroxidation and mitochondrial dysfunction cause irreversible tubular cell injury, highlighting ferroptosis as a pivotal mechanism in DKD progression [50]. Overall, these findings suggest that TECs are key targets in the pathophysiology of DKD.

### 3.2. Inflammatory Cell Death and Glomerular Injury

Podocytes are specialized epithelial cells of the glomerulus that are essential for maintaining the integrity of the glomerular filtration barrier. Loss of podocytes is a central feature in the pathogenesis of proteinuria and glomerulosclerosis [65,66]. In podocytes, pyroptosis (NLRP3/GSDMD-dependent) synergizes with necroptosis (RIPK3/MLKL-driven) and PANoptosis (TRAIL-mediated) to exacerbate cell loss. Notably, PANoptosis integrates all three pathways, with TRAIL triggering simultaneous caspase-8 (apoptosis), GSDMD (pyroptosis), and MLKL (necroptosis) activation. Pyroptosis not only leads to podocyte death but also contributes to the inflammatory milieu that exacerbates glomerular injury [16]. The activation of the NLRP3 inflammasome in podocytes promotes their death and loss, exposing the glomerular basement membrane (GBM) and resulting in proteinuria [67]. Although evidence for pyroptosis in podocyte injury is still emerging, it is clear that podocyte loss drives structural and functional alterations in the glomerulus, including glomerulosclerosis and tubulointerstitial fibrosis. The interplay between podocyte death and inflammatory signaling represents a potential therapeutic target for preserving podocyte health and preventing kidney disease progression [15,68].

Mesangial cells also play crucial roles in DKD pathophysiology [69]. Mesangial cell death in DKD is predominantly driven by pyroptosis and secondary necroptosis, with minimal ferroptosis involvement due to low iron susceptibility. Pyroptosis in mesangial cells triggered by NLRP3 inflammasome activation can lead to interleukin-1β (IL-1β) release, which contributes to mesangial matrix expansion and inflammation [70]. Chronic hyperglycemia induces cellular stress that further activates the NLRP3 inflammasome, amplifying IL-1β secretion and inflammatory responses [15]. Targeting pathways such as the NLRP3 inflammasome could mitigate inflammation and mesangial cell death-related renal damage [16].

Glomerular endothelial cells (GECs) are critical for filtration barrier function. Endothelial injury is an early event in DKD pathogenesis and can be precipitated by inflammatory processes [71], including pyroptosis. GECs exhibit mixed pyroptosis and NETosis-induced damage, where neutrophil-derived extracellular traps (NETs) exacerbate endothelial injury through TLR9 activation. Pyroptotic GEC death leads to the release of IL-1β and IL-18, aggravating inflammation and tissue injury [71]. IL-33 has also been implicated in endothelial inflammation [72]. Endothelial injury increases permeability and albumin leakage, which are hallmark signs of kidney damage, and predisposes patients to thrombosis within the renal microvasculature. Thinning of the glomerular capillary walls and vascular rarefaction further impair renal function.

### 3.3. Inflammatory Cell Death in Immune Cells

Inflammatory cell death in immune cells, particularly macrophages, plays a pivotal role in DKD progression36863097. Macrophages predominantly undergo pyroptosis (NLRP3/caspase-1-dependent) and NETosis (in polarized M1 subsets), amplifying renal inflammation via IL-1β and citrullinated histone release. Macrophages are central to the inflammatory response, and their activation can induce pyroptosis via inflammasome formation, caspase-1 activation, and GSDMD cleavage, leading to cell lysis and proinflammatory cytokine release [73]. NLRP3 inflammasome activation in macrophages exacerbates renal injury and fibrosis [16]. Interactions between macrophages and renal cells, such as podocytes and endothelial cells, form feedback loops that sustain inflammation. Chronic inflammation promotes immune cell recruitment, amplifying injury. Targeting macrophage activation and pyroptosis pathways may offer therapeutic opportunities in DKD [10].

In DKD, neutrophils primarily contribute through NETosis (PAD4-mediated), with minimal ferroptosis or necroptosis activity due to their short lifespan. NETs, which are formed during NETosis, are DNA–protein structures that play dual roles in host defense and pathology. In DKD, neutrophil infiltration into renal tissue increases NET formation, aggravating inflammation and promoting thrombosis. While NETs can trap pathogens and debris, they also contribute to tissue damage. Elevated IL-1β and IL-18 levels are associated with NET formation. Oxidative stress and mitochondrial dysfunction exacerbate NETosis under hyperglycemic conditions [15]. Targeting NETosis, for example, via DNase or anti-inflammatory agents, may mitigate renal inflammation and thrombosis in DKD [15].

## 4. Inflammatory Response and the Progression of Diabetic Kidney Disease

In addition to inflammatory cell death, the inflammatory response itself plays a crucial role in the pathogenesis of DKD, where hyperglycemia triggers a cascade of inflammatory processes that contribute to renal injury. Recent research has identified multiple inflammatory mediators and pathways involved in DKD, underscoring the importance of understanding these mechanisms to develop targeted therapies. Notably, the NLRP3 inflammasome has emerged as a key mediator of inflammation in DKD, promoting the secretion of proinflammatory cytokines such as IL-1β and IL-18, which exacerbates kidney damage [15]. Furthermore, the activation of inflammatory pathways can induce cellular stress responses that are highly relevant in DKD [9].

### 4.1. Central Role of Inflammatory Factors and Pyroptosis in DKD

Inflammatory factors play pivotal roles in DKD progression. Pyroptosis-related inflammatory mediators have been implicated in podocyte injury [10]. The NLRP3 inflammasome, a critical regulator of pyroptosis, is activated under hyperglycemic conditions, leading to increased IL-1β and IL-18 production, which further propagates inflammation and renal damage [63]. Targeting the NLRP3 inflammasome has been shown to mitigate renal inflammation and improve kidney function in diabetic models, suggesting that inhibiting pyroptosis could represent a novel therapeutic approach for DKD [74].

The interplay between oxidative stress and inflammation in DKD is significant, as oxidative stress amplifies the activation of inflammatory pathways, creating a vicious cycle that accelerates kidney damage [15]. The inhibition of inflammatory cytokines and the modulation of pyroptosis-related pathways have emerged as promising strategies to slow DKD progression [10]. Collectively, pyroptosis acts both as an initiator of renal inflammation and as a bridge linking cell injury to subsequent fibrosis, thereby positioning it as a central therapeutic target in DKD [16,63,75]. DAMPs are critical mediators of immune activation, particularly in the context of necroptosis. Key DAMPs, including high mobility group box 1 (HMGB1), adenosine triphosphate (ATP), and DNA, are released during necroptosis and act as potent activators of the immune system. These molecules bind to pattern recognition receptors (PRRs), such as TLRs and NOD-like receptors (NLRs), on immune cells, triggering inflammatory cascades. DAMP–TLR interactions initiate signaling pathways that promote the production of proinflammatory cytokines, including TNF-α, IL-6, and monocyte chemoattractant protein-1 (MCP-1). In DKD, DAMP-induced NLRP3 activation by HMGB1 and ATP further drives the maturation and release of IL-1β and IL-18, amplifying inflammation [15].

The activation of TLRs and NLRs by DAMPs is central to sterile inflammation. TLR binding triggers NF-κB activation, which induces proinflammatory cytokine production, whereas NLRP3 inflammasome activation cleaves procaspase-1, leading to IL-1β and IL-18 maturation. This coordinated TLR–NLR signaling promotes chronic inflammation in DKD, making these pathways attractive therapeutic targets [74]. Upon release, DAMPs drive cytokine synthesis, recruiting immune cells and sustaining inflammation. TNF-α can induce apoptosis in neighboring cells, IL-6 can act both pro- and anti-inflammatorily, and MCP-1 is essential for monocyte recruitment. Dysregulated cytokine production fuels persistent inflammation and tissue injury, underscoring the need to understand and modulate DAMP-driven responses [74].

### 4.2. From Inflammatory Cell Death to Fibrosis

Different inflammatory cell death modalities drive fibrosis through distinct molecular cascades: (1) pyroptosis releases IL-1β and IL-18, which activate fibroblasts via NF-κB-mediated TGF-β1 upregulation; (2) necroptosis liberates HMGB1, which binds to TLR4 on fibroblasts, stimulating collagen synthesis through Smad3 phosphorylation; and (3) ferroptosis (GPX4 inhibition) in tubular cells generates lipid peroxides (e.g., 4-HNE) that directly activate the fibroblast-to-myofibroblast transition via Nrf2/HO-1 axis disruption. Thus, inflammatory cell death not only aggravates inflammation but also directly promotes the initiation of fibrotic remodeling in DKD.

### 4.3. Amplification of Fibrosis Through Pyroptosis and DAMPs

Podocyte death via pyroptosis, necroptosis, and PANoptosis releases DAMPs (e.g., HMGB1), which, through TLR4/NF-κB signaling, sustain local inflammation and activate fibroblasts. This cascade leads to myofibroblast differentiation, extracellular matrix accumulation, and progressive structural remodeling. Persistent inflammation and NLRP3-driven pyroptosis form a self-reinforcing cycle that exacerbates DKD-associated fibrosis [10,63]. Given this interplay, targeting pyroptosis or modulating inflammatory responses could help prevent or reverse DKD-associated fibrosis [16].

### 4.4. Fibroblast Activation by Dying Cell-Derived Signals

The fibrotic microenvironment in DKD is shaped by cell death-specific factors: (1) Pyroptotic cells release IL-1β → induce epithelial-to-mesenchymal transition (EMT) in tubular cells by transforming growth factor beta/SMAD family member 3 (TGF-β/Smad3) and Snail1 upregulation; (2) Necroptotic cells release HMGB1 and ATP → activate NLRP3 in macrophages → secrete platelet-derived growth factor (PDGF) and connective tissue growth factor (CTGF); (3) Ferroptotic cells: Generate oxidized phospholipids → directly stimulate fibroblast proliferation through 12-lipoxygenase (LOX-12) activation.

Cytokines released from dying kidney cells, notably IL-1β and TGF-β, play crucial roles in fibroblast activation. IL-1β, which is released during pyroptosis, promotes fibroblast proliferation and extracellular matrix production [70]. TGF-β, a master regulator of fibrosis, drives fibroblast-to-myofibroblast differentiation, leading to collagen deposition and scar formation [9]. Moreover, macrophage recruitment under hyperglycemic conditions (notably M2 polarization) sustains fibrosis through PDGF and CTGF release, whereas EMT further expands the fibroblast pool under chronic inflammatory and hyperglycemic conditions. These combined processes accelerate glomerulosclerosis and tubulointerstitial fibrosis, highlighting multiple converging pathways that perpetuate renal scarring in DKD.

## 5. Therapeutic Targeting of Inflammatory Cell Death in DKD

**Figure 2:**
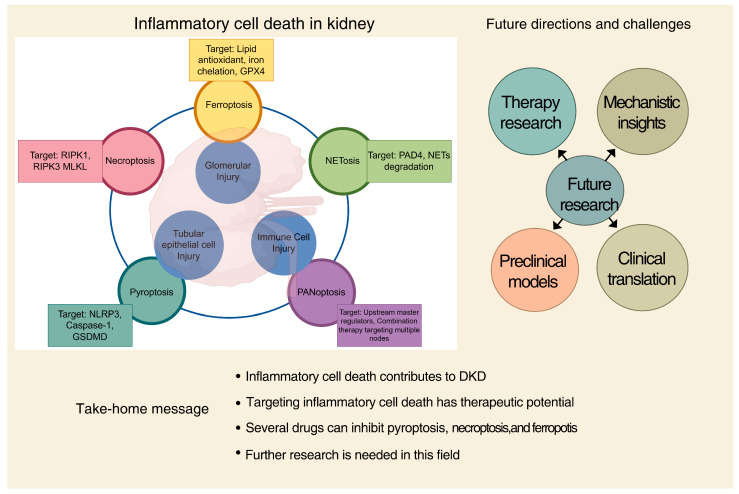
Therapeutic targeting of inflammatory cell death pathways in diabetic kidney disease (DKD) and future perspectives. By Figdraw (www.figdraw.com).

The central role of inflammatory cell death in DKD pathogenesis makes it a compelling therapeutic target. This section discusses the experimental and potential clinical strategies, organized by their molecular targets, and evaluates the evidence and challenges associated with each approach (Figure 2).

This schematic summarizes the key inflammatory cell death pathways involved in DKD and the key therapeutic strategies aimed at inhibiting various inflammatory cell death pathways implicated in diabetic kidney disease (DKD). The figure also highlights future research directions and challenges, including the need for mechanistic insights, combination therapies, and clinical translation. Key take-home messages emphasize the central role of inflammatory cell death in DKD pathogenesis, the promising therapeutic potential of its inhibition, and the necessity for further investigation.

### 5.1. Targeting the NLRP3 Inflammasome and Pyroptosis

Inhibition of the NLRP3 inflammasome represents one of the most promising strategies for mitigating pyroptosis-driven renal injury.

Mechanism: NLRP3 inhibitors (e.g., MCC950, OLT1177, and tranilast) prevent NLRP3 oligomerization and subsequent caspase-1 activation, thereby blocking GSDMD cleavage and the release of IL-1β and IL-18 [76,77,78,79,80].

Preclinical evidence: In STZ-induced diabetic rodents, MCC950 significantly reduces albuminuria, renal inflammation (IL-1β, IL-18), and fibrosis and preserves podocyte integrity [81]. A study further demonstrated that the protective effects of MCC950 are mediated in part by modulating mitochondrial dysfunction and reducing TXNIP expression in tubular cells [82]. OLT1177 similarly attenuated renal fibrosis and inflammation in db/db mice [83], whereas tranilast ameliorated renal damage by concurrently suppressing NLRP3 and oxidative factors [84].

Clinical Perspective: The efficacy of NLRP3 inhibition in heart failure and gout models supports its potential translatability. However, long-term safety regarding infection risk requires careful evaluation in future clinical trials.

### 5.2. Inhibiting Executioner Caspases

Directly targeting the executioners of pyroptosis offers a complementary strategy.

Mechanism: VX-765 is a potent and selective caspase-1 inhibitor that prevents the cleavage of pro-IL-1β and GSDMD [85,86].

Preclinical evidence: Administration of VX-765 to diabetic model mice markedly reduced the levels of active IL-1β and cleaved GSDMD, resulting in improved renal function, reduced macrophage infiltration, and attenuated podocyte injury. Its effects are also linked to the downregulation of the NF-κB and MAPK signaling pathways [87,88].

Clinical Perspective: While promising, caspase-1 has broad biological functions. The therapeutic window and potential off-target effects of its chronic inhibition need to be defined.

### 5.3. Targeting Necroptosis: RIPK1, RIPK3, and MLKL

Inhibiting the core necroptosis machinery can disrupt the cycle of TNF-α-driven inflammation.

Mechanism: Necrostatin-1 (Nec-1) inhibits RIPK1 kinase activity. GSK872 and GSK2982772 are selective inhibitors of RIPK3 and both RIPK1/RIPK3, respectively. These factors prevent the phosphorylation and activation of MLKL [89,90].

Preclinical evidence: Nec-1 treatment in diabetic mice reduces tubular cell death, inflammatory cytokine release (TNF-α, IL-6), and renal fibrosis [91]. GSK872 administration effectively suppressed MLKL phosphorylation, ameliorated renal inflammation, and improved the glomerular filtration rate in DKD models [92,93].

Clinical Perspective: The development of more specific and potent RIPK inhibitors is ongoing. Their utility may lie in combination therapies for patients with prominent inflammatory phenotypes [94,95].

### 5.4. Ferroptosis Inhibition: Scavenging Lipid Peroxides

Preventing iron-dependent lipid peroxidation protects susceptible tubular cells.

Mechanism: Ferrostatin-1 (Fer-1) and liproxstatin-1 (Lip-1) are potent radical-trapping antioxidants that halt the propagation of lipid peroxidation, independent of GPX4.

Preclinical evidence: Both Fer-1 and Lip-1 have shown remarkable efficacy in multiple DKD models [96,97]. These compounds rescue GPX4 activity, reduce the levels of lipid peroxidation products (4-HNE, MDA), and significantly alleviate tubular cell death and renal fibrosis. A 2022 study revealed that the benefit of Lip-1 is also associated with the restoration of mitochondrial health in proximal tubules [98].

Clinical Perspective: The pharmacokinetics of these first-generation compounds (e.g., poor solubility and stability) limit their clinical use. The development of next-generation ferroptosis inhibitors with improved drug-like properties is a major focus of current research (Table 2).

This table summarizes promising pharmacological inhibitors and agents that target key molecular components involved in pyroptosis, necroptosis, and ferroptosis. For each target, the corresponding representative inhibitor/agent, its mechanism of action, key protective effects observed in models of DKD, and relevant literature references are provided. The targeting of the NLRP3 inflammasome (e.g., MCC950 and OLT1177), executioners of pyroptosis (e.g., VX-765, which targets caspase-1), key regulators of necroptosis (e.g., Necrostatin-1, which targets RIPK1, and GSK872, which targets RIPK3), and the process of ferroptosis (e.g., ferrostatin-1 and liproxstatin-1) has been shown to ameliorate various pathological features of DKD, including inflammation, fibrosis, podocyte loss, and tubular injury. Multiple target agents, such as solasonine and β-sitosterol, demonstrate the therapeutic potential of simultaneously modulating multiple pathways.

### 5.5. Challenges in Clinical Translation and Future Perspectives

Despite promising preclinical data, several challenges must be overcome.

Pathway Redundancy and Crosstalk: A major hurdle is the redundancy between cell death pathways. Inhibiting one (e.g., necroptosis via RIPK3) may lead to compensation through another (e.g., increased pyroptosis). This underscores the need for multitarget inhibitors or rational combination therapies (e.g., an NLRP3 inhibitor with a ferroptosis inhibitor) to achieve robust efficacy [99,100].

Cell-Type-Specific Targeting: Systemic inhibition of innate immune pathways (e.g., NLRP3) carries a risk of immunosuppression. The development of kidney-targeted drug delivery systems (e.g., nanoparticles functionalized with renal tubule-targeting peptides) is crucial for enhancing on-target efficacy and minimizing systemic side effects [101,102].

Biomarker-driven personalized therapy: Not all patients respond to a given targeted therapy. Identifying predictive biomarkers (e.g., high urinary HMGB1 for necroptosis activity and elevated plasma IL-18 for pyroptosis) is essential for stratifying patients and personalizing treatment, ensuring that the right drug is used for the right patient.

Integration with Current Care: Future clinical trials should investigate whether these novel agents provide additive benefits when combined with the current standard of care, such as SGLT2 inhibitors or RAS blockers, which may themselves modestly modulate these inflammatory pathways.

Conclusion: Targeting inflammatory cell death represents a paradigm shift in DKD therapy. While clinical translation faces challenges, the concerted effort to develop targeted inhibitors, overcome biological redundancy and identify biomarker-defined patient subsets holds immense promise for halting the progression of DKD.

## 6. Conclusions

The complex interplay between inflammatory cell death and the pathogenesis of DKD highlights a crucial research domain with profound therapeutic implications. Through mechanisms such as pyroptosis and necroptosis, inflammatory cell death not only intensifies the inflammatory environment but also drives the loss of multiple renal cell types, thereby promoting the progressive fibrosis characteristic of DKD. This review underscores the essential role of these inflammatory pathways as amplifiers of disease progression, setting them apart from classical apoptotic mechanisms. A detailed understanding of inflammatory cell death is vital for advancing novel therapies for DKD, and a careful balance between diverse research perspectives—spanning cellular biology for clinical applications—must be maintained. Although classical apoptotic pathways have long dominated renal research, emerging evidence indicates that inflammatory forms of cell death represent distinct and independent pathological mechanisms that deserve dedicated investigation. Future research should seek to clarify the regulatory mechanisms underlying inflammatory cell death and their potential as therapeutic targets. Such efforts include investigating pharmacological agents capable of modulating these pathways to mitigate renal injury and inflammation. Moreover, translational research is essential to bridge laboratory findings with those of clinical practice, ensuring that novel therapies targeting inflammatory cell death can be effectively applied in patient care. In conclusion, the central role of inflammatory cell death in DKD progression represents a promising frontier in nephrology. A deeper understanding of these pathological processes, combined with multidisciplinary approaches that bridge basic research and clinical practice, will lay the foundation for transformative progress in the prevention and treatment of diabetic kidney disease. As the field continues to advance, carefully balancing diverse research perspectives will be critical for translating these insights into better outcomes for patients affected by this debilitating condition.

## Figures and Tables

**Table 1:** Characteristics of inflammatory cell death pathways in diabetic kidney disease (DKD).

Cell Death Type	Core Molecular Mediators	Key Effectors/DAMPs Released	Primary Renal Cellular Targets in DKD	Potential Biomarkers
Pyroptosis	NLRP3 inflammasome, Caspase-1/4/5/11, GSDMD	IL-1β, IL-18, GSDMD pores	Podocytes, Tubular epithelial cells, Macrophages	Plasma IL-18, Cleaved GSDMD (tissue)
Necroptosis	RIPK1, RIPK3, MLKL	HMGB1, ATP	Tubular epithelial cells, Podocytes	Urinary HMGB1, p-MLKL (tissue)
Ferroptosis	GPX4, FSP1, System Xc^−^, ACSL4	Lipid peroxides (e.g., 4-HNE)	Tubular epithelial cells	Plasma/Sermal lipid peroxides, 4-HNE (tissue)
NETosis	PAD4, Neutrophil Elastase, MPO	Citrullinated Histones, NETs (DNA fibers)	Glomerular endothelial cells	Circulating cf-DNA, MPO-DNA complexes
PANoptosis	Integrated molecular complex from Pyroptosis, Apoptosis, and Necroptosis	Combination of all above	Podocytes	Multianalyte panels (e.g., IL-18 + HMGB1)

**Table 2:** Therapeutic agents targeting inflammatory cell death pathways in diabetic kidney disease (DKD).

Target	Representative Inhibitor/Agent	Mechanism of Action	Key Effects in DKD Models
NLRP3	MCC950	Selective NLRP3 inflammasome inhibitor	IL-1β/IL-18; renal fibrosis; proteinuria; preserves podocytes
	OLT1177	NLRP3 inflammasome suppressor	Tubular injury; inflammation; attenuates glomerulosclerosis
	Tranilast	NLRP3 pathway inhibitor	Oxidative stress; macrophage infiltration; improves renal function
Caspase-1	VX-765	Caspase-1 inhibitor	GSDMD cleavage; pyroptosis; IL-1β; reduces podocyte injury
GSDMD	Necrosulfonamide	Blocks GSDMD pore formation	Pyroptosis-induced inflammation; protects tubular cells
RIPK1	Necrostatin-1 (Nec-1)	RIPK1 kinase inhibitor	Necroptosis; TNF-α-driven inflammation; fibrosis; podocyte loss
RIPK3	GSK872	RIPK3 kinase inhibitor	MLKL phosphorylation; necroinflammation; improves renal function
MLKL	Necrosulfonamide (NSA)	Covalently modifies MLKL to prevent oligomerization	Blocks necroptosis; reduces renal cell death
Ferroptosis (GPX4/ACSL4/SLC7A11)	Ferrostatin-1 (Fer-1)	Radical-trapping antioxidant	Lipid peroxidation; rescues tubular cell death; fibrosis
	Liproxstatin-1 (Lip-1)	Inhibits lipid peroxidation	Ferroptosis markers; improves glomerular filtration
Multi-target	Solasonine	Modulates Nrf2/NLRP3 axis	Pyroptosis in podocytes; oxidative stress
	β-Sitosterol	Suppresses NLRP3 activation	Renal inflammation; GSDMD cleavage

## Data Availability

No new data were created or analyzed in this study. Data sharing is not applicable to this article.

## Abbreviations

The following abbreviations are used in this manuscript:

ACSL4	Acyl-CoA Synthetase Long Chain Family Member 4
AGEs	Advanced Glycation End-Products
ASC	Apoptosis associated speck like proteins
ATP	Adenosine triphosphate
cGAS	cyclic GMP–AMP Synthase
CTGF	Connective Tissue Growth Factor
DAMPs	Damage-Associated Molecular Patterns
DKD	Diabetic Kidney Disease
dsDNA	Double-Stranded DNA
EMT	Epithelial-to-Mesenchymal Transition
EndMT	Endothelial-to-Mesenchymal Transition
ESRD	End-Stage Renal Disease
Fer-1	Ferrostatin-1
FTH1	Ferritin Heavy Chain 1
GBM	Glomerular Basement Membrane
GECs	Glomerular Endothelial Cells
GLP-1	Glucagon-Like Peptide-1
GLP-1RA	Glucagon-Like Peptide-1 receptor agonist
GPX4	Glutathione Peroxidase 4
GSDMD	Gasdermin-D
HMGB1	high mobility group box 1
IL-1β	Interleukin 1-beta
IL-33	Interleukin-33
LOX-12	12-Lipoxygenase
MCP-1	Monocyte Chemoattractant Protein-1
NETs	Neutrophil Extracellular Traps
NF-κB	Nuclear Factor kappa B
NLRP3	NOD-, LRR-, and pyrin domain-containing protein 3
NLRs	NOD-Like Receptors
Nrf2	nuclear factor erythroid 2–related factor 2
NSA	Necrosulfonamide
PAD4	Peptidyl arginine deiminase 4
PAMPs	Pathogen-Associated Molecular Patterns
PDGF	platelet-derived growth factor
PRRs	pattern recognition receptors
RAS	Renin–Angiotensin System
RIPK1	Receptor-Interacting Protein Kinase 1
RIPK3	Receptor-Interacting Protein Kinase 3
SGLT2	Sodium–Glucose Cotransporter 2 Inhibitors
SIRT1	Sirtuin 1
SLC7A11	Solute Carrier Family 7 Member 11
Smad3	SMAD Family Member 3
STAT3	Signal transducer and activator of transcription 3
STING	Stimulator of Interferon Genes
System Xc^−^	acystine/glutamate antiporter system
TECs	Tubular Epithelial Cells
TFR1	Transferrin Receptor 1
TGF-β	Transforming growth factor beta
TLR2	Toll like receptor 2
TLR9	Toll-like receptor 9
TLRs	Toll-Like Receptors
TNF	Tumor necrosis factor
TNFR1	Tumor necrosis factor receptor 1
TRAIL	TNF-Related Apoptosis-Inducing Ligand
ZDSD	Zucker Diabetic-Sprague Dawley
